# A Cross-Sectional Quantitative Study on Sexual and Reproductive Health Knowledge and Access to Services of Arab and Kurdish Syrian Refugee Young Women Living in an Urban Setting in Lebanon

**DOI:** 10.3390/ijerph18189586

**Published:** 2021-09-11

**Authors:** Rayan Korri, Guenter Froeschl, Olena Ivanova

**Affiliations:** 1 Munich Medical Research School (MMRS), Medical Faculty of the University of Munich (LMU), 80336 Munich, Germany; 2 Division of Infectious Diseases and Tropical Medicine, Medical Centre of the University of Munich (LMU), 80802 Munich, Germany; froeschl@lrz.uni-muenchen.de (G.F.); Olena.ivanova@lrz.uni-muenchen.de (O.I.); 3 German Center for Infection Research (DZIF), Partner Site Munich, 80802 Munich, Germany

**Keywords:** young women, refugee health, vulnerability, sexual and reproductive health, public health, urban setting, forced migration, Lebanon, Syria, cross-sectional survey

## Abstract

Since data on the sexual and reproductive health (SRH) of young refugee women living in urban settings in Lebanon are particularly scarce, we aim through this exploratory study to assess the SRH knowledge and access to services of Arab and Kurdish Syrian refugee young women living in Bourj Hammoud. From January to March 2020, a cross-sectional survey was conducted among 297 Syrian Arab and Kurdish participants and aged 18–30 years old. It was found that participants coming from Syrian urban areas or who completed an education above secondary level have higher overall knowledge on SRH issues. Only a total of 148 out of the 297 participants (49.8%) knew a health facility in Bourj Hammoud that provides SRH services and among them 36.4% did not know which type of services are available there. The Syrian refugee young women’s access to SRH services is inadequate due to different obstacles. The overall knowledge level on different SRH topics is limited. The context of multiple crises in Lebanon should be taken into consideration when delivering future SRH services.

## 1. Introduction

During the 21st century, the world experienced a considerable increase in the number of individuals who were forced to migrate due to conflicts, civil disorder, expulsion, and assault. The number of refugees and asylum seekers escalated from 17 to 34 million between 2000 and 2020, with half of it being composed of women and girls [1]. In 2021, 25% of the global refugees come from Syrian Arab Republic. Most of Syrian refugees are hosted by neighboring countries, where 19 out of 20 live in urban regions [2]. Lebanon is one of those countries, which hosts the worldwide highest number of refugees per capita [3]. The Lebanese Government did not allow the creation of camps as formal settings for Syrian refugees, who as a consequence became scattered across the country and inhabiting rented rooms, apartments, garages, and informal tented settlements (ITSs) [4,5,6]. Furthermore, 89% of Syrian refugee families in Lebanon live below the survival minimum expenditure basket (SMEB) defined in the country and experience distressing living conditions [7,8].

The armed conflict in Syria, which continues since 2011, did not only create a public health catastrophe within the country, but also critical public health challenges in the neighboring countries which received refugees [9]. The Lebanese healthcare system is inequitable, in large shares privatized, and is based on out-of-pocket payments [10,11]. With the arrival of Syrian refugees, the system became additionally strained with an excessive demand since its coverage had also to cope with disadvantaged Lebanese individuals, Lebanese citizens returning from Syria, Palestinian refugees that had to give up their settlements in Syria, and in general with an already pre-existing refugee population in the country consisting mainly of Palestinian refugees that arrived in the aftermath of the conflicts of 1948 and 1967 [12]. As a result, the refugees were left with restricted, insufficient, and hard to access services [10,13].

Previous research showed that young people and women experience additional hardships during conflicts and emergencies that lead to health deterioration [14,15]. Women and girls living in humanitarian settings tend to suffer from poor sexual and reproductive health (SRH) outcomes, which put them at increased risk of morbidity and mortality [16,17,18,19]. Furthermore, Syrian refugee women in Lebanon experience difficulties when seeking SRH services because of high service costs, absence of female healthcare providers, and discriminatory attitudes from providers [20,21]. A needs assessment has shown that only 32% of Syrian women within the reproductive age in Lebanon consider SRH services easily accessible, while 38% think that these services are practically unavailable and 17% are unaware that these services even exist [22]. Moreover, a situation analysis conducted in 2013 by the United Nations Population Fund (UNFPA) on youths in Lebanon, who are affected by the Syrian crisis, found that only 31% of refugee participants received health services, and 56% of those found the services satisfactory. The analysis also showed that Syrian youths had insufficient knowledge on SRH issues. For instance, only 45% of refugee youths self-declared knowledge of contraceptive methods, of whom one quarter indicated withdrawal as one of the methods [23].

Since data on the SRH of young refugee women living in urban settings in Lebanon are particularly scarce, the general aim of this exploratory study is to assess the SRH status of Arab and Kurdish Syrian refugee young women living in Bourj Hammoud. Its specific objective is to determine the knowledge of refugee young women on SRH issues such as sexually transmitted infections (STIs) and contraceptive methods on one hand and their access to SRH services such as ever visited health facility in Lebanon and healthcare provider characteristics on the other hand. The agenda of the International Conference on Population and Development (ICPD) and the *Inter-Agency Field Manual on Reproductive Health in Humanitarian Settings* (IAFM) by the Inter-Agency Working Group on Reproductive Health in Crises (IAWG) form the framework of the study [17,24]. Its objective and results are in line with the Sustainable Development Goal (SDG) Number Three—*ensure healthy lives and promote well-being for all at all ages*—which also encompasses the necessity to advance reproductive, maternal, and child health [25]. This study complements a qualitative research, conducted previously by our team, in which qualitative insights on knowledge and experiences around SRH of Syrian girls aged between 13 and 17 years also living in Bourj Hammoud were provided [26]. Our findings are aimed at improving and focusing health promotion activities on SRH in refugee populations.

## 2. Materials and Methods

### 2.1. Study Setting

According to the United Nations High Commissioner for Refugees (UNHCR), 8141 Syrian refugees registered the industrial area of Bourj Hammoud as their place of residence [27]. The area has a history of accommodating refugees since the 1920s, where Armenians arrived after surviving genocide and escaping expulsion by Ottomans [28]. In the present, individuals with lower socio-economic status—including Lebanese citizens, Syrian, Palestinian, and Iraqi refugees, and migrant workers—reside in Bourj Hammoud [29,30]. The suburb, which is one of the most heavily inhabited in the Middle East, suffers from inadequate living conditions such as unsatisfactory infrastructure, hygiene conditions, and supply of electricity and clean drinking water [28,31].

### 2.2. Study Design

We employed a cross-sectional survey to explore the SRH knowledge of refugee young women and their experiences in accessing services. The questionnaire consisted of five sections: demographic characteristics (e.g., age, ethnic group, level of education); displacement characteristics (e.g., year of fleeing, reason of fleeing, and duration of stay in Bourj Hammoud); individual agency in displacement (e.g., head of household, healthcare decision making power); SRH knowledge (e.g., sources of information, knowledge on contraceptive methods and STIs); experiences in accessing SRH services (e.g., ever visited health facility in Lebanon for SRH services, healthcare provider characteristics); and experiences of pregnancy (e.g., number of pregnancies and antenatal care visits in Lebanon). The questionnaire’s different parts were developed based on two validated tools: Reproductive Health Assessment Toolkit for Conflict-Affected Women, CDC, 2007 [32] and Adolescent Sexual and Reproductive Health Toolkit for Humanitarian Settings, UNFPA and Save the Children, 2009 [33]. The questionnaire was designed in English, translated into Arabic, and piloted before the start of the data collection.

### 2.3. Sample Participants

We calculated a sample size of 297 and managed to enroll 305 Syrian refugee young women. The sample size was determined based on Cochran’s (1963) formula for cross sectional studies with a precision of 5% and a confidence level of 95% [34]. The prevalence of self-claimed knowledge of contraceptive methods among Syrian refugee girls and young women from previous studies was adopted [23,35]. Snowball sampling was used to recruit participants. When conducting research that includes hidden groups such as vulnerable refugee communities, snowball sampling method is found to be the most suitable [36,37].

Five different snowball starting points were applied through five Syrian female community gatekeepers. In order to avoid a homogenous sample and to ameliorate representation, gatekeepers belonging to various age and ethnic groups, coming from different areas in Syria, and having distinct socio-economic characteristics (e.g., education level and monthly income) were chosen. Additionally, we allowed only a limited number of participants from each resulting chain [38,39]. The efficiency of engaging gatekeepers in the recruitment procedure for research on sensitive topics that involve refugee communities as participants has been previously reported [40,41]. The inclusion criteria of respondents were: bearing Syrian nationality, belonging to Arab or Kurdish ethnic groups, age between minimum 18 and maximum 30 years, and date of arrival to Lebanon only after the start of the armed conflict in Syria (set at 15 March 2011). Eight questionnaires were excluded from the study, since their corresponding participants moved to Lebanon before 15th of March 2011. Since snowball sampling was implemented, there is no means to estimate the number of individuals who refused to participate in the study.

### 2.4. Data Collection

Data collection was carried out from January to March 2020 by the first author—a Lebanese female doctoral researcher, who is an Arabic native speaker. Data collection was completed using a tablet computer, on which the questionnaire was programmed employing the Magpi^®^ application. Data were collected one-on-one in a private environment, either in the participants’ or in the gatekeepers’ apartments.

### 2.5. Data Analysis

Data were analyzed using IBM SPSS Statistics version 27.0.(International Business Machines Corporation, New York, NY, USA) A descriptive presentation of the results of the questionnaire is given for continuous variables that are non-normally distributed through interquartile range (IQR) and medians. Since none of the variables were normally distributed, standard deviation (SD) and means were not calculated. Tests of associations were conducted for categorical variables using Fisher’s exact test, since the study’s sample size, and in consequence size of cells, is considered small [42,43]. The Chi-square test was used to check for significant differences between proportions across categories (SRH service categories). A threshold of significance was set at 0.05. No data were missed.

In order to evaluate the overall knowledge of participants on SRH issues, an unweighted score was generated for every participant based on her knowledge on different SRH topics, as reported by Ivanova et al. [44] in a comparable study in Uganda: STIs, symptoms of STIs, methods of contraception, and danger signs of pregnancy. Each of these elements were assessed through a scale from zero to three. After combining the evaluation from the four elements and getting the final average score, the overall knowledge on SRH issues was described as following: low (average score ≤ 1), medium (average score between 1 and 2), and high (average score ≥ 2) [44].

### 2.6. Ethical Considerations

Before administering the structured questionnaire, the researcher explained the aim and relevance of this study to participants, who also learned about their right to participate on a voluntary basis and to withdraw their participation at any time. Written Arabic informed consent was received from participants. In case of illiterate participants, oral Arabic informed consent was received in the presence of a witness. The Institutional Review Boards of Rafik Hariri University Hospital in Lebanon and the Faculty of Medicine at Ludwig-Maximilians-Universität in Munich, Germany, provided the ethical approvals for this study (Project Nr. 19-552).

## 3. Results

### 3.1. Demographic and Displacement Characteristics of Participants

**Figure 1:**
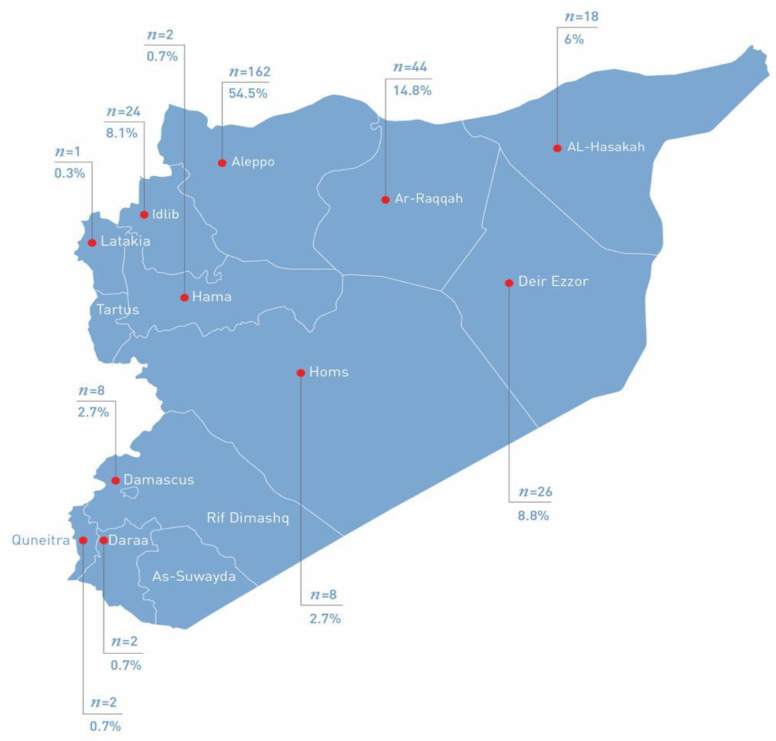
Distribution of participants (*n* = 297) by governates of origin in Syria.

Two hundred and ninety-seven (297) young women participated in the survey. The median age of participants was 25 years (IQR: 21–29), with 72.7% of them being Syrian Arab and 27.3% being Syrian Kurdish. The young women arrived from 11 out of 14 Syrian governates (administrative districts in Syria), with the majority (*n* = 162; 54.5%) coming from Aleppo and only one participant coming from Latakia. The distribution of participants between the different governates is presented in Figure 1. The vast majority of participants were married (*n* = 268; 90.2%). A total of 51.2% (*n* = 128) of young women had acquired an education below secondary level and 48.8% (*n* = 145) of them completed an education above secondary level. A total of 89.9% (*n* = 267) of the participants had a monthly income, while the rest of the young women (*n* = 30) were not receiving any income for the last three to five months, since the beginning of the economic crisis in Lebanon. Most of the young women (*n* = 131; 44.1%) received a monthly income of USD 100-399. More than half of the participants (*n* = 159; 53.5%) lived in urban areas before arriving to Lebanon, while the rest lived in rural areas (*n* = 138; 46.5%). The largest proportion of participants indicated to be living as refugees between 5 and 10 years, be it in Lebanon (*n* = 174; 58.6%), or more precisely in Bourj Hammoud (*n* = 137; 46.1%). Fear and security concern was the most frequent given reason for fleeing (*n* = 217; 73.1%), followed by economic difficulties (*n* = 35; 11.8%), reunification with a husband (*n* = 31; 10.4%), and lack of daily necessities (*n* = 14; 4.7%). The median number of individuals sharing the same place of residence with the participants was 5 (IQR: 4–8), with 2 being the median number of adults (≥18 years of age) and 3 the median number of children (<18 years of age) in residence. The detailed demographic and displacement characteristics of the participating Syrian refugee young women are presented in Table 1.

### 3.2. Individual Agency in Displacement

For most of the participants (*n* = 241; 81.1%), the husband was the head of the household. That was followed by a relative (*n* = 27; 9.1%) or a parent (*n* = 16; 5.4%). Only 13 out of the 297 young women were themselves the head of their household. Most of the participants were financially dependent on their husbands (*n* = 250; 84.2%), while some others depend on family members (*n* = 33; 11.1%). Only 14 out of the 297 young women were financially independent through a job they had in Bourj Hammoud. None of the participants depended on a direct support by UNHCR or a non-governmental organization (NGO). We asked the young women about the decision maker in their household when it comes to their healthcare, mobility, work and participation in workshops, and physical appearance. Most of participants had their own last say when it comes to decisions regarding their mobility (*n* = 115; 38.7%), ability to work or participate in workshops (*n* = 115; 38.7%), and physical appearance (*n* = 167; 56.2%). However, the biggest percentage of young women (*n* = 102; 34.3%) had to make a joint decision with their husband/partner for issues related to their own healthcare. In case the decisions were not made independently or jointly, the husband/partner or another relative (e.g., mother or mother-in-law) was the final decision maker. An overview of the results is presented in Table 2. We also asked the young women about the final decision maker regarding the household’s daily purchases. Most participants (*n* = 99; 33.3%) were the decision makers on that regard, followed by the husband/partner (*n* = 86; 29%), a joint decision with the husband/partner (*n* = 66; 22.2%), and another relative (*n* = 46; 15.5%). When having a closer look only among married participants (*n* = 268), we also found that the majority of the young women made independent decisions regarding their mobility (*n* = 106/268; 39.5%), work and participation in workshops (*n* = 106/268; 56%), physical appearance (*n* = 150/268; 56.6%), and household’s daily purchases (*n* = 91/268; 40%), but had to make a joint decision with their husband or partner regarding their own health (*n* = 102/268; 38%).

### 3.3. SRH Knowledge and Sources of Information

The participants approached multiple people when seeking information on SRH issues. The majority of them reached to female relatives other than their mother and sister (*n* = 92), followed by friends (*n* = 77), partner or husband (*n* = 65), mother (*n* = 64), sister (*n* = 53), doctor or nurse (*n* = 40), educational provider (*n* = 2), and male relative (*n* = 1). Only eight participants wished to reach to someone else. A total of 134 out of 297 (45.1%) participants also looked for similar information through different online channels, such as YouTube (*n* = 89/134; 66.5%), Google (*n* = 35/134; 26%), and social media (e.g., Facebook and Instagram; *n* = 10/134; 7.5%).

We evaluated the knowledge of participants on different SRH topics: STIs, symptoms of STIs, methods of contraception, and danger signs of pregnancy. A total of 161 out of the 297 participants (54.2%) were not able to identify any STI, while 61 out of the 297 participants (20.5%) were not able to name any symptom associated with STIs. Among participants who were knowledgeable about these two issues, HIV/AIDS (*n* = 136/136; 100%) and genital itching (*n* = 167/236; 70.7%) were the most STI and related symptom mentioned. The vast majority of Syrian refugee young women (284; 95.6%) knew at least one method of contraception. Birth control pills (*n* = 268/284; 94.3%), IUD (*n* = 266/284; 93.6%), and withdrawal (*n* = 257/284; 90.5%) were the three most identified methods by the knowledgeable young women. Most of the participants (*n* = 231; 77.7%) could name at least one danger symptom which is associated with pregnancy. Vaginal bleeding (*n* = 199/231), intense abdominal pain (*n* = 123/231), and fever (*n* = 116/231) were the three most known symptoms. An overview of the young women’s SRH knowledge is presented in Table 3 and a detailed demonstration of that knowledge on the four topics is enclosed in supplementary (Tables S1–S4).

In bivariate analyses using Fisher’s exact test, no association was found neither between the overall knowledge on SRH and age (*p*= 0.387) nor between the same overall knowledge and duration of stay in Lebanon (*p*= 0.90). However, the overall knowledge on SRH issues was found to be associated with the type of setting in which the Syrian refugee young women lived before being displaced to Lebanon (*p* < 0.001) in addition to their level of education (*p* < 0.001). Participants coming from Syrian urban areas were more likely to have a higher overall knowledge on SRH issues compared to participants who inhabited rural areas. Furthermore, Syrian refugee young women who acquired an education below secondary level tended to have a poorer knowledge on SRH topics compared to the ones who completed an education above secondary level.

### 3.4. Access to SRH Services

**Figure 2:**
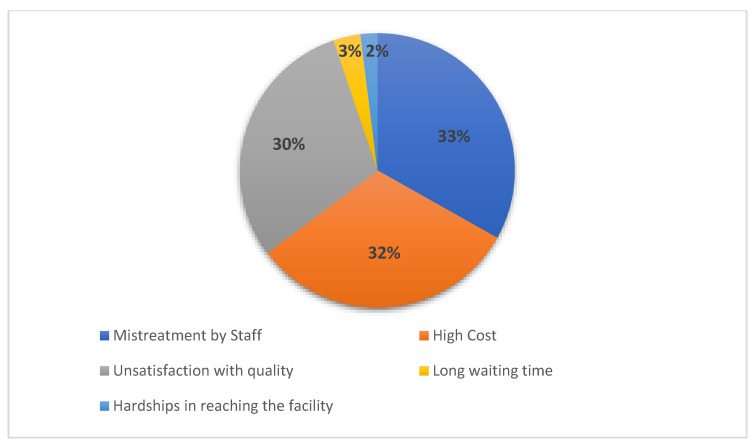
Reasons for not returning to the health facility.

We assessed the medical check-ups and procedures received by the participants during their stay in Lebanon. The majority of the young women had at least one general check-up by a gynaecologist (*n* = 233; 78.5%) and one blood test (*n* = 197; 66.3%). Only 27.6% (*n* = 82) of them had at least one check-up by a general practitioner and 15.5% (*n* = 46) of them received at least one vaccination. Very few participants (*n* = 26; 8.8%) reported that they had at least one pap smear during their stay in Lebanon. A total of 28 out of the 297 participants (9.4%) did not receive any medical check-up or procedure during their displacement to Lebanon. Most of the participants (*n* = 240; 80.8%) visited at least once a health facility in Lebanon to receive SRH services. They knew about it through a friend (*n* = 108/240; 45%), a relative (*n* = 106/240; 44.2%), a healthcare provider (*n* = 15/240; 6.2%), or an NGO worker (*n* = 9/240; 3.7%). Only two young women could not remember how they came to know about the facility and its offered services. The last visit for the majority of participants was for receiving pregnancy care and delivery (*n* = 156/240; 65%), followed by STIs treatment and counselling (*n* = 33/240; 13.75%), family planning services (*n* = 21/240; 8.75%), and education or counselling regarding different SRH topics (*n* = 11/240; 4.6%). In addition, 19 out of the 240 participants (7.9%) visited lately a health facility to receive other SRH services such as hormonal therapy and infertility treatment. All the young refugee women talked to a medical doctor, except one participant who talked to a midwife. A female healthcare provider delivered the needed SRH service for the majority of participants (*n* = 174/240; 72.5%). The young women described the healthcare provider as friendly and helpful (*n* = 195/240; 81.3%), unfriendly and disrespectful (*n* = 22/240; 9.2%), friendly but unhelpful (*n* = 19/240; 7.9%), and unexperienced (*n* = 4/240; 1.6%). The biggest percentage of young women (*n* = 177/240; 73.7%) would return again to the health facility. The reasons for not returning for the rest of them are presented in Figure 2.

When asked about their preferred sex of service provider, 52.2% (*n* = 155) of the total participants favoured females and 1.3% (*n* = 4) favoured males. A noticeable percentage of the young women (*n* = 138; 46.5%) did not have any preference.

**Figure 3:**
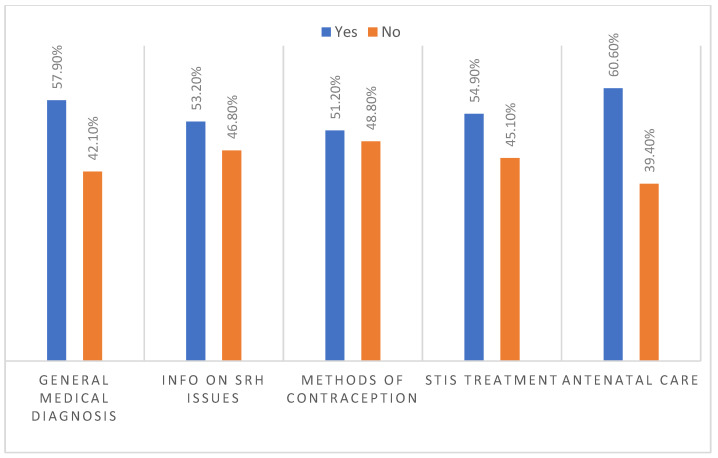
Awareness of the participants on available and accessible health services in Bourj Hammoud and its neighbouring urban areas.

Only half of the participants (*n* = 148; 49.8%) knew a health facility in Bourj Hammoud that provides SRH services. There was no significant association between familiarity with a SRH care provider on the one hand and number of years lived in Bourj Hammoud, bearing head of household position, income level or healthcare decision making power on the other hand. When being asked about the type of services available at the facility, 36.4% (*n* = 54/148) of the young women did not have any answer. We examined the awareness of the participants regarding the availability and accessibility of five different categories of SRH services in Bourj Hammoud and its neighbouring urban areas. More than half of the participating young women knew where to access health services that are related to general medical diagnosis, information on SRH issues, methods of contraception, STIs treatment, and antenatal care. The results are presented in Figure 3. We tested for significant differences in existing knowledge on service availability between the five SRH categories using the Chi-square test. That allowed us to check in case the difference is statistically significant. The participants indicated significantly higher knowledge on the service categories of general medical diagnosis (*p* = 0.006) and antenatal care (*p* < 0.001), in contrast to information on SRH issues (*p* = 0.270), methods of contraception (*p* = 0.685), or STIs treatment (*p* = 0.92).

### 3.5. Experiences of Pregnancy

A total of 236 out of the 297 young refugee women (79.5%) reported their experience of pregnancy during their stay in Lebanon. The median number of pregnancies was two (IQR: 1–3). Furthermore, 89 out of the 236 participants (37.7%) stated to have suffered a miscarriage, where 18 reported more than one miscarriage. Almost all participants who experienced pregnancy in Lebanon (*n* = 227/236; 96.2%) received antenatal care. The majority of them (*n* = 172/227; 75.8%) had three or more antenatal visits during their last pregnancy. For the same last pregnancy in Lebanon, 53.8% (*n* = 127/236) of the young women wanted to become pregnant then, 33.9% (*n* = 80/236) preferred to wait longer before becoming pregnant, 11.9% (*n* = 28/236) did not want to become pregnant anymore, and one participant had no response to the question.

## 4. Discussion

This cross-sectional quantitative study assessed the general SRH status of Arab and Kurdish Syrian refugee young women living in Bourj Hammoud, Lebanon, and determined their knowledge on SRH issues and access to SRH services. Its findings show the Syrian refugee young women limited overall SRH knowledge and insufficient access to needed services within their urban setting of residence specifically and in Lebanon as a host country generally.

In our study, a low number of participants (46 out of 297) received immunization. A similar finding was reported in 2013 among pregnant Syrian women living in different Lebanese urban areas, where only 8.0% of women were vaccinated against tetanus [45]. Immunization of mothers is key to prevent maternal, neonatal, and young children morbidity and mortality [46,47]. Additionally, vaccination of girls and young women against the human papillomavirus (HPV) prohibits cervical cancer. In 2018, almost 90% of the deaths caused by this disease took place in low- and middle-income countries [48]. A very limited number of participating young women (8.8%) had at least one pap smear during their displacement to Lebanon. According to the Centers for Disease Control and Prevention (CDC), women having the age between 21 and 29 years should receive one pap smear every three years in case of a normal test. This important screening tool, which detects malignant and premalignant lesions of the cervix, allows an early diagnosis of cervical cancer [49].

Among the participants who visited a healthcare facility in Lebanon, 65% accessed SRH services that are related to pregnancy care and delivery. This was also observed in an assessment conducted in Lebanon only a year after the start of the Syrian conflict, where 59.7% of the displaced women never visited a gynaecologist if not for pregnancy care or delivery [22]. The insignificant variance in the presented numbers throughout the prolonged Syrian crisis highlights the need to increase the awareness among refugees on all available SRH services at reduced prices on one hand and their locations of availability on the other hand. Additionally, it is necessary to extend the awareness through refugee’s different networks, including social media [50]. Syrian refugee young women living in Bourj Hammoud reported different barriers that limit their access to available SRH services. Mistreatment by staff, high cost, poor quality of services, long waiting times, far distances, and unaffordable means of transport were the obstacles mentioned by participants. Comparable barriers are observed in Syrian refugee populations in Jordan and Turkey [51,52], in addition to other displaced populations such as refugee adolescent girls in the Nakivale refugee settlement in Uganda [44]. Surprisingly, and in contrary to other studies on Syrian refugee women in Jordan and Turkey [35,53,54], the sex of the healthcare provider was not named as a barrier to SRH services access. Differently, a recognizable percentage of participants (46.5%) did not have any preference concerning the sex of service provider. Some of the participants described healthcare providers by disrespectful, unhelpful, and unexperienced, and expressed their unsatisfaction with the quality of received SRH services. These reports reflect the participants’ major concerns regarding the skills of the healthcare provider on one hand and the sufficiency of SHR services on another hand, and not in respect to the sex of healthcare provider. It is true that more than half of the participants knew where to go to receive health services in Bourj Hammoud and its neighbouring urban areas, however the percentage of Syrian young women who do not know where to have this access is still considered elevated (ranged between 39.4 and 48.8%). This emphasizes once more time the necessity to expand the awareness among refugees on available SRH services-such as receiving information on SRH issues, methods of contraception, STIs treatment, and antenatal care. Although there was no significant association between familiarity with a SRH care facility and the healthcare decision making power, it is essential to further examine the effect of the women’s dependent decisions on their own SRH status, especially that the majority of participants (34.3%) had to make a joint decision with their husband or partner.

The participants had limited overall knowledge on four SRH topics: STIs, symptoms of STIs, methods of contraception, and danger signs of pregnancy. Their knowledge on at least one contraception method was the highest (95.6%), followed by at least one symptom of STIs (79.5%), at least one danger sign of pregnancy (77.7%), and at least one STI (45.8%). Although neither age nor duration of stay in Lebanon were found to affect the participants’ overall level of knowledge, this knowledge seems to depend on Syrian refugee young women’s education level and type of setting in which they lived before being displaced to Lebanon. According to McKay, women cannot have a SRH care decision making power if they are not provided with precise and comprehensive information in the first place [55]. Women from lower social class receive less information because of the healthcare providers’ assumption that they are not able to comprehend scientific knowledge [55]. In this study, HIV was found to be the most known type of STI. A similar finding was reported among Syrian refugee mothers in Jordan [56]. The considerable national and international awareness campaigns on HIV, which is not the case for other STIs, could be the cause behind that [56]. Interestingly, participants had acceptable knowledge on STIs symptoms but could not identify most of the STIs. This could be due to the tightly connected social networks of refugees, through which Syrian women exchange information that focus on sharing personal experiences, specially that friends, relatives, and partners or husbands were the main sources of information for the vast majority of participants.

Although almost all participants were knowledgeable of at least one method of contraception, 45.8% of women who experienced pregnancy in Lebanon had low or no desire for their last pregnancy. Thus, there is a gap between the level of knowledge on contraceptive methods on one hand and the actual use of these methods on another hand. Some studies reported a restricted level of contraceptive use within the population of Syrian refugee women in Lebanon, which ranged from 42.3 to 65.5% [22,45]. A qualitative study on Syrian refuge women in Turkey found that participants had sufficient knowledge on modern contraceptive methods but could not identify their efficiency [57]. Therefore, the very high level of knowledge on contraception methods among the participants of this study can be a result of an over-reporting, where women are only aware of the methods’ names but not of their functions and effectiveness.

SRH started to be incorporated in the humanitarian responses and programs that tackle different types of crises since the 1990s [58,59]. These programs, and regardless of their application level, should be designed based on the particular context of each country in which they will be implemented [60]. In case of extended crises, such as the Syrian armed conflict that has been lasting for the past 10 years, healthcare systems become fragile which negatively affect the health status of women [13,61,62]. It is essential to describe and recognize the present complex and multi-layered Lebanese context, in order to better understand its impact on the well-being of Syrian refugees in general and the SRH of Syrian refugee women in specific. Lebanon is experiencing several complex crises since October 2019: economic breakdown, political unsteadiness, the COVID-19 pandemic, and the explosion at the Port of Beirut on the 4th of August 2020 [63]. These crises were added to the vulnerable conditions of refugees as a result of the conflict in Syria [63,64].

The economic crisis, which started in October 2019 and its effects were slightly witnessed during the data collection of our study, is considered one of the three worst economic crises worldwide since the mid-19th century [63]. A drastic increase in the unemployment rate, one of the crisis’ consequences, was reflected in the findings of the study, where 30 participants have not received any income since October 2019. The protracted financial and political crisis hinders the providing of crucial public services, including health services, and thus impairs the well-being of individuals [63]. According to Médecins Sans Frontières (MSF), the increase in the inflation rate to 133% by November 2020 distressed Lebanese citizens as well as refugees and obstructed their capability to access satisfactory healthcare services [64]. Furthermore, the economic crisis pushed at least half of the Lebanese population under the national poverty line [63]. In an already inequitable, stretched, and remarkably privatized healthcare system, the crisis generates additional obstacles to access healthcare services and cause the health deterioration of already vulnerable groups [64]. These populations will have to put first their family’s life saving needs such as food and shelter before their own SRH needs [65]. In a phone survey conducted by the World Food Program (WFP), 36% of households reported barriers in accessing health care between November and December 2020, a percentage that increased from 25% between July and August 2020 [63].

The Lebanese public healthcare system was also stressed due to the increasing number of COVID-19 patients starting of spring 2019. An assessment conducted by the Interagency Sexual and Gender-based Violence (SGBV) before the 4th of August 2020 to study the pandemic’s effect on the level of SGBV throughout the country, found that 51% of the participating women and girls, including Syrian refugees, feel less safe and only 30% of them are still accessing health services [66]. Finally, the blast at the Port of Beirut impaired six main hospitals in addition to 23 primary health care centers and caused the loss of medical supplies in different types of healthcare settings: primary, secondary, and tertiary [67]. Since this study’s data collection phase took place between January and March 2020, its results do not show the serious effects of the Lebanese multiple crises. All these events might contribute to further worsening the SRH of Syrian refugee women and are expected to continue in doing so.

The combined effect of the several crises on Syrian refugee young women’s SRH status, knowledge, and access to available services should be investigated in depth in order to complement the new needs of women who are experiencing an increased vulnerability. The evaluation of the existing services and programs should also be performed to determine their level of suitability and sufficiency vis-à-vis to the necessary requirements to avert poor SRH outcomes, specially that no clear plan is being drafted on the governmental level to resolve the different crises.

We recognize the different limitations of this study. First of all, the researcher was not able to always assert the reported age of participants based on available official documents. Second, the self-reporting conducted by participants might have caused over- or under-reporting, especially with the effect of social desirability bias. Moreover, the study’s sample is non-representative, since no random sampling method was applied. However, and since the aim of our exploratory study is to have insights into the SRH of refugee young women living in an urban setting, which is overlooked in research, representation was not the preference [68,69]. The study on a sensitive topic such as SRH, participants’ anxiety about the research intentions, and restrictions when building connections and trust within the Syrian refugee community living in Bourj Hammoud presented challenges when recruiting participants and thus limited women’s participation and representation. Finally, the cross-sectional type of the study did not allow an investigation of the changes in the participants’ SRH knowledge and access to services at different points in time during their displacement to Lebanon.

## 5. Conclusions

Syrian refugee young women residing in Bourj Hammoud have restricted access to SRH services and unsatisfactory overall knowledge on different SRH topics. Thus, it is necessary to expand the awareness among refugee women on all affordable and available SRH services in urban settings and not to only focus on refugees’ maternal health. Provision of information on variety of different SRH issues and treatment of STIs are some of those services that are still inadequate. Furthermore, an effective intervention targeting these challenges should always be designed according to the context of the setting in which it will be implemented. Such a design will assure constructive outputs, where refugee women’s SRH status is enhanced.

This study provides valuable primary data on the SRH knowledge and access to services among young refugee women living in an urban setting, which makes them a hard-to-reach group. The findings could guide future research on specific SRH components of Syrian refugee women in Lebanon in specific and of other young refugee populations in the extended Middle East and North Africa (EMENA) countries in general. Such research is highly needed in Lebanon in order to shape the work of national, international, governmental, and non-governmental institutions that support this target group through SRH services, especially within a context of multiple crises that are expected to further deteriorate the SRH status of Syrian refugee women and lead to urgent poor SRH outcomes.

## Figures and Tables

**Table 1:** Demographic and Displacement Characteristics of Participants.

	Number (*n* = 297)	Percentage (%)
**Individual Characteristics**		
**Age**		
18–24 Years	145	48.8
25–30 Years	152	51.2
**Ethnic Group**		
Arabs	216	72.7
Kurds	81	27.3
**Educational Level**		
Never attended school	24	8.1
Primary	128	43.1
Secondary	90	30.3
Tertiary	36	12.1
University	13	4.4
Vocational training	6	2
**Marital Status**		
Single	17	5.7
Engaged	3	1
Married	268	90.2
Divorced	7	2.4
Widowed	2	0.7
**Income**		
No income	30	10.1
<USD 100	13	4.4
USD 100-399	131	44.1
USD 400-600	107	36
>USD 600	16	5.4
**Displacement Characteristics**		
**Lived Before Fleeing Syria**		
In a village	138	46.5
In a city	159	53.5
**Reason of Fleeing**		
Security concerns/fear	217	73.1
Lack of daily necessities	14	4.7
Economic difficulties	35	11.8
Reunification with husband	31	10.4
**Live in Lebanon for**		
Less than 1 year	14	4.7
1–4 years	109	36.7
5–10 years	174	58.6
**Live in Bourj Hammoud for**		
Less than 1 year	38	12.8
1–4 years	122	41.1
5–10 years	137	46.1
**Head of Household**		
Self	13	4.4
Husband	241	81.1
Parent	16	5.4
Other Relative	27	9.1

**Table 2:** Final decision maker in household on matters that concern the young women.

	Matter	Healthcare	Mobility	Work/Participation in Workshops	Physical Appearance
Decision Maker					
Self		85	115	115	167
Husband/partner		84	106	103	66
Joint decision with husband/partner		102	50	54	49
Other relative		26	26	25	15
Total		297	297	297	297

**Table 3:** Sexual and Reproductive Health Knowledge of Participants.

	Number (*n* = 297)	Percentage (%)
**Knowledge of STIs**		
0	161	54.2
1	83	27.9
2	31	10.4
3 or more STIs	22	7.4
**Knowledge of STIs Symptoms**		
0	61	20.5
1	37	12.5
2	64	21.5
3 or more symptoms	135	45.5
**Knowledge of Contraception**		
0	13	4.4
1	11	3.7
2	14	4.7
3 or more methods	259	87.2
**Knowledge of Pregnancy’s Danger Signs**		
0	66	22.2
1	53	17.8
2	68	22.9
3 or more signs	110	37

## Data Availability

The dataset and materials used in this study are available from the first author on reasonable request.

## Supplementary Materials

The following are available online at https://www.mdpi.com/article/10.3390/ijerph18189586/s1, Table S1. Identified STIs among Knowledgeable Participants (*n* = 136); Table S2. Identified STIs Symptoms among Knowledgeable Participants (*n* = 236); Table S3. Identified Methods of Contraception among Knowledgeable Participants (*n* = 284); Table S4. Identified Danger Signs of Pregnancy among Knowledgeable Participants (*n* = 231).
