# Supplementary material for: A Cross-Sectional Quantitative Study on Sexual and Reproductive Health Knowledge and Access to Services of Arab and Kurdish Syrian Refugee Young Women Living in an Urban Setting in Lebanon
Source: Int J Environ Res Public Health. 2021 Sep 11;18(18):9586. doi: 10.3390/ijerph18189586 (PMC8471977; doi:10.3390/ijerph18189586)
Supplement: Supplementary file 1 [file ijerph-18-09586-s001.zip › ijerph-1318592-supplementary.pdf]

International Journal of
Environmental Research and Public Health

MDPI

Supplementary:

Additional file 1. SRH knowledge of Syrian refugee young women on four SRH topics

Table S1. Identified STIs among Knowledgeable Participants (n = 136).

	Number	Percentage (%)
HIV/AIDS	136	100
Hepatitis B	45	33
Chlamydia	2	1.5
Genital Herpes	16	11.8
Genital Warts	12	8.8
Gonorrhea	8	5.9
HPV	10	7.3

Table S2. Identified STIs Symptoms among Knowledgeable Participants (n = 236).

	Number	Percentage (%)
Abdominal pain	66	28
Green or curd-like vaginal discharge	131	55.5
Foul-smelling discharge	123	52.1
Burning pain during urination	164	69.5
Redness in genital area	65	27.5
Genital ulcers or sores	66	28
Genital itching	167	70.7
Pain during intercourse	52	22
Loss of weight	6	2.6
Yellow eyes and skin	3	1.3
Hard to get pregnant	17	7.2

Table S3. Identified Methods of Contraception among Knowledgeable Participants (n = 284).

	Number	Percentage (%)
Birth Control Pills	268	94.3
IUD	266	93.6
Male Condom	194	68.3
Implant	100	35.2
Patch	91	32
Injection	105	37
Emergency Pill	0	0
Sterilization	167	58.8
Calendar	148	52.1
Withdrawal	257	90.4

Table S4. Identified Danger Signs of Pregnancy among Knowledgeable Participants (n = 231).

	Number	Percentage (%)
Feeling very weak or tired	45	19.5
Severe abdominal pain	123	53.2
Vaginal bleeding	199	86.1
Fever	116	50.2

Swelling of hands and face	93	40.3
Headache	16	6.9
Blurred vision	15	6.5
Sudden water breaking	8	3.5
Vomiting and Dizziness	13	5.6
Infections	4	1.7
Hormonal Instability	1	0.4
Pale Face	1	0.4
Absence of foetal movement	1	0.4
Low position of the foetus	2	0.8
